# The health behaviour status of teenage and young adult cancer patients and survivors in the United Kingdom

**DOI:** 10.1007/s00520-019-04719-y

**Published:** 2019-05-29

**Authors:** G. Pugh, R. Hough, H. Gravestock, A. Fisher

**Affiliations:** 1 Centre for Sports & Exercise Medicine, William Harvey Research Institute, Queen Mary University of London, Mile End Hospital, Bancroft Road, London, E1 4DG UK; 2 Department of Behavioural Science & Health, University College London, 1-19 Torrington Place, London, WC1E 6BT UK; 3 Department of Haematology, University College London Hospital, London, UK; 4 CLIC Sargent, No.1 Farriers Yard, L77-85 Fulham Palace Road, London, W6 8JA UK

**Keywords:** Physical activity, Diet, Tobacco use, Alcohol use, Survivorship

## Abstract

**Purpose:**

The primary aim of this study was to investigate the health behaviour status of teenage and young adult (TYA) cancer patients and survivors; the secondary aim was to determine if TYA cancer patients and survivors health behaviour differs to general population controls.

**Methods:**

Two hundred sixty-seven young people with cancer (*n* =83 cancer patients receiving active treatment: *n* =174 cancer survivors, 57.1% >1 year since treatment completion) and 321 controls completed a health and lifestyle questionnaire which included validated measures of physical activity (PA) (Godin Leisure Time Exercise Questionnaire), diet (Dietary Instrument for Nutrition Education, DINE), smoking status, and alcohol consumption (AUDIT-C).

**Results:**

General population controls and cancer survivors were more likely to meet current (PA) recommendations (*p* <0.001) than TYA cancer patients undergoing treatment (54.8% vs 52.3% vs 30.1%, respectively). Less than 40% of young people with cancer and controls met fat intake, sugar intake, fibre intake or current fruit and vegetable recommendations. TYA cancer survivors were more likely to report binge drinking than controls (OR=3.26, 95% CI 2.12–5.02, *p* <0.001). Very few young people with in the study were current smokers. The majority of TYA cancer patients and survivors reported a desire to make positive changes to their health behaviour.

**Conclusion:**

Consideration should be given to whether existing health behaviour change interventions which have demonstrated positive effects among the general TYA population could be adapted for young people with cancer.

## Introduction

It is estimated that of the 16,630 teenagers and young adults (TYA) living with and beyond cancer in the UK, more than two-thirds will suffer a physical or psychosocial health problem as a result of their cancer diagnosis and treatment [1–3]. Nevertheless, there is growing evidence that positive health behaviours (particularly physical activity) may improve physical and psychosocial health outcomes among young people with cancer, both during and after cancer treatment [4–6]. As such, there is increasing recognition that TYA cancer patients and survivors should be provided with health behaviour change support throughout the cancer continuum [7].

However, very little is known regarding the current health behaviour status of TYA cancer patients or survivors and whether their lifestyles differ to the general population. Of the few studies which have investigated the health behaviours of young people with cancer, the majority have been conducted in the USA and predominantly explored the health behaviour status of long-term survivors of a cancer diagnosed during childhood [8–14]. Data from these studies suggest TYA-aged cancer patients and survivors have low levels of physical activity, consume relatively poor diets, often struggle with weight gain, and engage in health-risk behaviours such as smoking and alcohol consumption. No data on the health behaviour status of TYA cancer patients and survivors in the UK has been published within the last decade [15].

Understanding the health behaviour status of TYA cancer patients and survivors is an important step in informing our understanding of how cancer affects the lifestyles of young people and how behaviour change interventions may be best tailored to meet their needs. Similarly, it is important to understand if young people with cancer have made, or perceive the need to make, positive changes to their lifestyle following their cancer diagnosis. Such data will provide insight into whether behaviour change interventions for young people with cancer are warranted.

The purpose of this study was to investigate if young people with cancer meet current lifestyle guidelines set by the Children’s Oncology Group and determine the extent to which their health behaviour differs to general population controls.

## Methods

### Design

TYA cancer patients, TYA cancer survivors and general population TYAs were invited to complete a health and lifestyle questionnaire based on an on-going large-scale health behaviour survey of adult breast, prostate and colorectal cancer survivors ‘ASCOT’ being conducted by our research group [16]. Both young people with cancer and general population TYAs had the option to complete the questionnaire in either online or in paper format.

### Participants and recruitment

As per the clinical age brackets set by the National Health Service (NHS) [17] and the National Cancer Institute [18] definition of cancer survivor, any young person between the age of 13 and 25 years who had been diagnosed with any type of cancer at any point in their lifetime was eligible to complete the health and lifestyle survey. This included young people aged 13–25 years currently undergoing cancer treatment and those who had been diagnosed with cancer during their childhood (aged 0–12 years). TYA cancer patients (those undergoing active cancer treatment at the time of the survey) and survivors were recruited via University College Hospital, London and project partners CLIC Sargent (a UK cancer charity for children and young people). General population controls were recruited online through schools, higher education establishments, social media, youth networks or websites directed towards young people in two recruitment waves. Ethical approval was obtained from University College London Research Ethics Committee reference 6206/001 and London Hampstead Research Ethics Committee reference 15/LO/0764.

### Measures

Participants were asked to report their age, gender, height, weight, highest level of educational attainment, marital status, current living arrangement and ethnicity. Young people with cancer were also asked to report their cancer diagnosis, date of diagnosis, current treatment status and any health problems (other than cancer) they suffer from.

A modified version of the Godin Leisure Time Exercise Questionnaire (GLTEQ) was used to assess physical activity. Participants were asked to report the frequency and duration of mild, moderate and strenuous exercise they engage in over typical week. The GLTEQ has been used in similar studies of TYA cancer survivors [19, 20] and has demonstrated reliability and validity within the oncology research setting [21]. The modifications to the original measure were minor and involved the addition of a single item to collect information regarding the average duration (in minutes) of each exercise intensity. This modification is common with over 80% of published articles using the GLETQ in oncology research reporting a similar small amendment to the original questionnaire [21]. An adapted version of the Dietary Instrument for Nutrition Education (DINE) food frequency questionnaire (FFQ) was used to assess fibre, fat, red meat intake, processed meat intake and sugar intake. Original items were adapted to be relevant to adolescents and young adults. A two-item consumption measure was used to assess fruit and vegetable intake [22–24]. Smoking status was determined using questions taken from the Health Survey for England [25]. Participants were asked to report if they currently smoke cigarettes and if so, how many per day. Smokers were asked to report if they ever tried to quit smoking before their cancer diagnosis and if they have tried to quit smoking since their cancer diagnosis. Those who reported not smoking were asked to report if they had ever smoked regularly in the past. Frequency and quantity of alcohol consumption was assessed using three items taken from the Alcohol Use Disorders Identification Test Consumption (AUDIT-C) scale [26]. Participants were asked to report their perception of whether they should change their health behaviour. Young people with cancer were asked if they had attempted to make changes to their lifestyle since their cancer diagnosis.

### Statistical analyses

Age, ethnicity, treatment status and number of health problems were dichotomised for the main analyses. Individual data on weight status, physical activity, diet, alcohol consumption and smoking were scored and dichotomised according to whether young people were meeting current World Cancer Research Fund and Children’s Oncology Group lifestyle recommendations (Table 1). The scoring procedure and cut-offs applied to each health behaviour are as outlined Supplementary File A. Self-reported height and weight were used to calculate BMI before classifying participants into obese, overweight, healthy weight and underweight categories. Descriptive statistics were produced to determine the proportion of participants meeting current health behaviour guidelines and the proportion of young people who report making changes to their lifestyle following their cancer diagnosis. Statistical comparisons between the three groups of participants: TYA cancer patients, TYA cancer survivors, and controls were made using chi-squared tests and logistic regression analysis for categorical variables and non-parametric Mann-Whitney tests for continuous variables. Multivariable models were adjusted for age and gender. Little’s missing completely at random (LMCAR) test was performed to evaluate the patterns of missing data within each measure of health behaviour. Available case analysis was carried out as for all measures of health behaviour in each group of participants, any residual missing data points were deemed to be missing completely at random (MCAR) as there was no significant systematic difference (*p* >0.005) between missing and observed values. Only data from wave 1 of general population recruitment was available for analysis.

**Table 1:** Lifestyle guidance for teenage and young adult cancer survivors (Children’s Oncology Group, 2008)

Health behaviour	Guidance
Physical activity	Check with healthcare team before starting an exercise plan or taking part in a new sport or recreational activities For adults—engage in moderate physical activity >30 min/day for >5 days per week For children and adolescents—engage in >60 min of moderate to vigorous physical activity for >5 days per week
Diet	Choose a variety of foods from all the food groups (grains, vegetables, fruits, oil, milk, meat and beans) Use the steps to a healthier you guide to develop a well-balanced diet and activity plan (www.mypyramid.gov)
Fruit/veg	Eat >5 servings of fruit and veg per day, including citrus fruits and dark green and deep yellow vegetables When drinking juice, choose 100% fruit or vegetable juice and limit to 4 oz. per day
Milk/dairy	Choose low-fat milk and dairy products
Meat	Limit intake of red meat and substitute with fish, poultry and beans When eating meat, select leaner or smaller portions
Fibre	Eat plenty of high-fibre foods, such as whole grain breads, rice, pasta and cereals
Fat	Decrease the amount fat in meals by baking, broiling or boiling foods Limit fried or high-fat foods
Sugar	Limit refined carbohydrates, including pastries, sweetened cereal, soft drinks and sugar
Salt intake	Avoid salt cured, smoked, charbroiled and pickled foods
Alcohol	Limit alcoholic drinks to <2 drinks per day for men and <1 drink per day for women
Smoking	Do not smoke
Sun safety	Limit the amount of time in the sun especially between 10 am and 2 pm Regularly using sunscreen with a sun protection factor of 15 or more Cover up in the sun and do not actively try and tan

## Results

### Participant characteristics

From the original sample (*n* =295 young people with cancer; *n* =370 wave 1 general population controls), 83 TYA cancer patients, 174 TYA cancer survivors and 321 general population TYAs provided complete data on all health behaviours. Participant characteristics are displayed in Table 2**.** TYA cancer patients and survivors were predominantly female (*n* =46, 55.4% and *n* =109, 62.9%, respectively), white British (*n* =63, 75.9% and *n* =134, 77.0%, respectively) and living at home with their immediate family (*n* =64, 77.1% and *n* =125, 71.8%). Most young people with cancer (*n* =33, 39.8%, TYA cancer patients, *n* =76, 43.7% TYA cancer survivors) reported being in full-time education. 37.3% (*n* =31) of TYA cancer patients reported being unable or too ill to work or study.

**Table 2:** Participant demographic and health characteristics

	TYA cancer patients* n =83% (*n*)	TYA cancer survivors* n =174% (*n*)	General population TYAs *n* =321% (*n*)
Age (mean ± SD)	19±3.06	20.0±2.78	17±3.1
Gender			
Females	55.4 (46)	62.9 (109)	77.6 (249)
Males	44.6 (37)	37.4 (65)	22.4 (72)
Educational status**			
GSCE/standard grade/school cert	65.1 (54)	68.4 (119)	48.9 (157)
Vocational qualifications (e.g. NVQ1 + 2)	18.1 (20)	11.5 (20)	2.5 (8)
A-level/higher school cert	38.6 (32)	52.9 (92)	14.3 (46)
Bachelor degree	8.4 (7)	18.4 (32)	9.3 (30)
Masters/PhD/PGCE	0 (0)	5.2 (9)	5.3 (17)
Still studying	41.0 (34)	33.9 (59)	62.3 (200)
No formal qualifications	4.8 (4)	1.1 (2)	2.2 (7)
Other	8.4 (7)	5.7 (10)	3.1 (10)
Employment status**			
Employed full-time	6.0 (5)	21.8 (38)	5.9 (19)
Employed part-time	14.5 (12)	22.4 (39)	7.2 (23)
Self-employed	0 (0)	1.7 (3)	1.6 (5)
Unemployed	4.8 (4)	5.7 (10)	8.1 (26)
Full-time education	39.8 (33)	43.7 (76)	77.3 (248)
Part-time education	6.0 (5)	4.6 (8)	0.9 (3)
Unable or too ill to work/study	37.3 (31)	13.2 (23)	0.3 (1)
Voluntary work	9.6 (8)	6.9 (12)	6.2 (20)
Living arrangement			
Alone	2.4 (2)	6.9 (12)	5 (16)
With my partner	10.8 (9)	9.2 (16)	4 (13)
With immediate family	77.1 (64)	71.8 (125)	80.1 (257)
Other family	3.6 (3)	0.6 (1)	1.9 (6)
With friends	4.8 (4)	11.5 (20)	7.8 (25)
Residential care	1.2 (1)	0 (0)	0.9 (3)
Ethnicity			
White British	75.9 (63)	77.0 (134)	41.1 (132)
White Irish	1.2 (1)	1.1 (2)	1.2 (4)
Black African	4.8 (4)	2.9 (5)	6.5 (21)
Black Caribbean	1.2 (1)	1.1 (2)	1.2 (4)
Indian	2.4 (2)	1.7 (3)	8.1 (26)
Bangladeshi	1.2 (1)	2.9 (5)	4.4 (14)
Pakistani	1.2 (1)	2.9 (5)	3.1 (10)
Chinese	0 (0)	0 (0)	4.4 (14)
Mixed White and Black African	0 (0)	0.6 (1)	1.6 (5)
Mixed White and Black Caribbean	2.4 (2)	0.6 (1)	1.2 (4)
Mixed White and Asian	1.2 (1)	0.6 (1)	3.7 (12)
Other	6.0 (5)	8.0 (14)	21.8 (70)

*Data on treatment status missing for 6 participants

**Where percentages do not equal 100%, this was due to participants selecting all that applied

General population controls had an average age of 17 years (SD=3.1) were predominantly female (*n* =249, 77.6%), in full-time education (*n* =248, 77.3%) and living at home with their immediate family (*n* =257, 80.1%). GP-TYAs were significantly younger than young people with cancer (mean difference 2.89, *p* <0.0001) and were more likely to be female (*X*^2^ (1, *n* =584)=22.66, *p* <0.001) and be from a non-white British ethnic background.

### Health characteristics

Table 3 outlines the cancer and treatment characteristics of TYA cancer patients and survivors. The most common cancer diagnoses were haematological malignancies (*n* =154, 59.2%), bone tumours (*n* =26, 10%) and soft tissue sarcomas (*n* =22, 8.5%). Average age at diagnosis was 16.5 years (SD=4.43). The majority of respondents (*n* =146, 56.1%) had finished cancer treatment and with more than half reporting that they were between 1 and 5 years from treatment completion (56.1% *n* =79). Young people with cancer were more likely to report one or more health problems than controls (82% vs 37%, *p* =0.001). The most common health difficulties among TYA cancer patients and survivors were extreme fatigue and mental health problems. The proportion classed as being either overweight or obese was significantly greater among young people with cancer than the general population (30.4% vs 9.6%; *p* <0.001).

**Table 3:** Cancer and treatment characteristics of TYA cancer survivors

	TYA cancer patients *n* =83% (*n*)	TYA cancer survivors *n* =174% (*n*)	TYA cancer survivors total *n* =267% (*n*)
Cancer diagnosis			
Lymphoma	22.9 (19)	31.5 (62)	31.5 (81)
Leukaemia	34.9 (29)	24.1 (42)	27.6 (71)
Bone tumour	7.2 (6)	10.9 (19)	9.7 (25)
Soft tissue sarcoma	15.7 (13)	5.7 (10)	8.9 (23)
CNS tumour	10.8 (9)	6.9 (12)	8.2 (21)
Germ cell tumour	0 (0)	5.2 (9)	3.5 (9)
Carcinoma	3.6 (3)	4.6 (8)	4.3 (11)
Melanoma	2.4 (2)	1.1 (2)	1.6 (4)
Other	4.8 (4)	8.0 (14.0)	7 (18)
Age at diagnosis (mean ± SD)	17.0±4.1	16.2±4.5	16.4±4.4
0–12 years	10.7 (8)	15.1 (25)	12.6 (33)
13–18 years	45.3 (34)	54.8 (91)	48.9 (127)
19–24 years	44.0 (33)	30.1 (50)	32.2 (84)
Missing data	9.6 (8)	4.6 (8)	6.2 (16)
Treatment*			
Surgery	56.9 (33)	57.4 (70)	57.2 (103)
Radiotherapy	51.8 (29)	46.9 (58)	50.3 (87)
Chemotherapy	94.6 (70)	97.5 (156)	96.6 (226)
Hormone therapy	5 (2)	8.7 (8)	7.6 (10)
Active surveillance	5 (2)	8.9 (8)	7.7 (10)
None	2.6 (1)	1.1 (1)	1.6 (2)
Not sure	5 (2)	1.1 (1)	2.4 (3)
Other	12 (10)	13.7 (24)	13.6 (36)
Time since treatment**			
<3 months from finishing treatment	–	13.2 (23)	–
4–11 months since finishing treatment	–	19 (33)	–
1–5 years since finishing treatment	–	46 (80)	–
>5 years since finishing treatment	–	5.7 (10)	–
On active surveillance	–	12.1 (21)	–
I don’t know	–	0.6 (1)	–
Missing data	–	3.4 (6)	–
			General population controls *n* =321% (*n*)
Health problems	% (*n*)	% (*n*)	% (*n*)
Osteoporosis	0 (0)	4 (7)	0.3 (1)
Diabetes	4.8 (4)	1.7 (3)	0.6 (2)
Asthma	10.8 (9)	8.6 (15)	13.4 (43)
Irregular heart rhythm	7.2 (6)	5.7 (10)	1.9 (6)
Extreme fatigue	31.3 (26)	29.9 (52)	6.2 (20)
Mental health problems	16.9 (14)	20.1 (35)	14.3 (46)
Lung disease	0 (0)	1.1 (2)	0.3 (1)
Arthritis	1.2 (1)	2.3 (4)	0.6 (2)
Any other heart trouble	1.2 (1)	1.7 (3)	0.9 (3)
Another cancer	2.4 (2)	1.1 (2)	0.3 (1)
Sensory impairment	1.2 (1)	5.2 (9)	1.2 (4)
Other	34.1 (28)	35.1 (61)	7.8 (25)
Weight classification			
Obese (BMI>30)	13.3 (11)	12.1 (21)	2.5 (8)
Overweight (BMI 25–29.9)	18.1 (15)	18.4 (32)	6.5 (21)
Healthy weight (BMI 18.5–24.9)	50.6 (42)	56.3 (98)	51.4 (165)
Underweight (BMI <18.5)	7.2 (6)	4.6 (8)	19 (61)
Missing data	10.8 (9)	8.6 (15)	20.6 (66)

*Where percentages do not equal 100%, this was due to participants selecting all that applied

**Data from those off treatment only (*n* =174)

### Health behaviour status

Table 4 presents the proportion of TYA cancer patients and survivors meeting current health behaviour recommendations and adjusted and unadjusted odds ratios for the association between treatment status and health behaviour. TYA cancer survivors were more likely to meet current physical activity recommendations (*p* <0.001) than those undergoing treatment (30.1% vs 52.3%, respectively); however there was no significant difference between TYA cancer survivors and general population controls (52.3% vs 54.8%, respectively). Less than 40% of either young people with cancer or general population controls were meeting recommendations for fat intake, sugar intake and fibre intake. In contrast, a high proportion (>80%) of participants in all groups met current recommendations for red meat intake of no more than 500 g per week. Less than a quarter met current fruit and vegetable recommendations of more than five portions per day. There was no significant difference in the dietary intake between TYA cancer patients and survivors. In comparison to general population controls, young people with cancer were significantly less likely (*p* <0.005) to meet fat intake, sugar intake and processed meat guidelines.

**Table 4:** Adjusted and unadjusted odds ratios for the association between treatment status and health behaviour

	Proportion meeting guidelines % (*n*)	Unadjusted		Adjusted^a^	
		Odds ratio (95% CI)	Odds ratio (95% CI)	Odds ratio (95% CI)	Odds ratio (95% CI)
Physical activity (*n* =577)					
GP-TYAs	54.8 (176)	1		1	
TYA-CS	52.3 (91)	0.35 (0.21, 0.59)	1	0.21 (0.81, 0.54)	1
TYA-CP	30.1 (25)	0.89 (0.62, 1.29)*	0.39 (0.22, 0.68)*	0.54 (0.21, 1.36)*	0.38 (0.21, 0.68)**
Fat intake (*n* =440)					
GP-TYAs	24.5 (78)	1		1	
TYA-CS	25.3 (44)	1.48 (0.83, 2.64)	1	3.41 (1.08, 10.77)*	1
TYA-CP	25.3 (21)	1.41 (0.90, 2.21)	0.80 (0.45–1.41)	3.33 (1.05, 10.55)*	0.781 (0.44, 1.38)
Fibre intake (*n* =521)					
GP-TYAs	39.3 (126)	1		1	
TYA-CS	26.4 (46)	1.77 (1.17, 2.69)*	1	1.62 (0.99, 2.67)*	1
TYA-CP	28.9 (24)	1.68 (0.98, 2.88)*	0.87 (0.47–1.59)	1.55 (0.86, 2.81)	0.949 (0.52, 1.71)
Sugar intake (*n* =460)					
GP-TYAs	38.9 (125)	1		1	
TYA-CS	32.2 (56)	1.91 (1.25, 2.90)*	1	1.84 (1.14, 2.95)**	1
TYA-CP	25.3 (21)	2.78 (1.58, 4.91)**	1.47 (0.80–2.71)	2.71 (1.47, 4.97)**	1.444 (0.78, 2.66)
Red meat intake (*n* =545)					
GP-TYAs	82.2 (264)	1		1	
TYA-CS	85.1 (148)	0.56 (0.29, 1.08)	1	0.93 (0.21, 4.08)	1
TYA-CP	81.9 (68)	1.04 (0.50, 2.13)	1.84 (0.78–4.32)	1.59 (0.39, 6.49)	1.882 (0.79, 4.45)
Processed meat (*n* =550)					
GP-TYAs	52.6 (169)	1		1	
TYA-CS	39.1 (68)	1.63 (1.11, 2.40)*	1	5.01 (1.93, 13.01)**	1
TYA-CP	33.7 (28)	2.18 (1.30, 3.64)*	1.33 (0.76, 2.32)	6.06 (2.32, 15.84)**	1.195 (0.66, 2.13)
Fruit and veg (*n* =527)					
GP-TYAs	25.2 (81)	1		1	
TYA-CS	15.5 (27)	1.73 (0.90, 3.31)	1	2.11 (0.69, 6.43)	1
TYA-CP	15.7 (13)	1.62 (0.99, 2.65)	1.06 (0.51, 2.20)	2.15 (0.68, 6.75)	0.961 (0.45, 2.02)
Smoking (*n* =533)					
GP-TYAs	86.0 (276)	1		1	
TYA-CS	79.9 (139)	1.95 (0.91, 4.18)	1	2.1 (0.94, 4.68)	1
TYA-CP	79.5 (66)	1.04 (0.46, 2.36)	0.53 (0.19, 1.44)	1.1 (0.49, 2.73)	0.533 (0.19, 1.46)
Alcohol consumption (units) (*n* =490)					
GP-TYAs	80.4 (82)	1		1	
TYA-CS	77.9 (109)	0.94 (0.39, 2.25)	1	0.92 (0.36, 2.35)	1
TYA-CP	86.4 (51)	0.22 (0.02, 1.68)	0.23 (0.02, 1.90)	0.19 (0.02, 1.58)	0.209 (0.02, 1.73)
Binge drinking (*n* =523)					
GP-TYAs	45.1 (46)	1		1	
TYA-CS	29.3 (41)	3.06 (2.03, 4.61)**	1	3.26 (2.12, 5.02)**	1
TYA-CP	50.8 (30)	1.1 (0.68, 2.02)	0.38 (0.21, 0.69)	1.26 (0.72, 2.2)	0.404 (0.21, 0.74)**

GP-TYAs, general population teenage and young adults; TYA-CS, teenage and young adult cancer survivors completed active treatment; TYA-CP, teenage and young adult cancer patients undergoing treatment

*p <0.05; **p <0.005

^a^OR adjusted for age and gender

Approximately one-third of TYA cancer patients and survivors (*n* =18, 31.8%) and general population controls (*n* =76, 34.9%) aged between 13 and 17 years reported under-age drinking. Among young adult participants (those aged 18–24 years at the time of the survey), most participants (>80%) were meeting the recommendations of no more than two alcoholic drinks per day. More than half of participants were classed as binge drinkers. TYA cancer survivors were more likely to report binge drinking than controls (OR=3.26, 95% CI 2.12–5.02, *p* <0.001). Very few young people with cancer or controls were current smokers. Those who reported smoking smoked an average of 8.6±3.17 and 9±1.9 cigarettes per day (young people with cancer and controls, respectively). A significantly larger proportion (*p* <0.005) of controls (*n* = 41, 15.9%) reported smoking in the past when compared to TYA cancer patients and survivors (*n* =19, 7.2%).

### Perception of current health behaviour

As displayed within Table 5, most young people with cancer and controls felt they should do more physical activity and over half felt they should have a healthier diet. Approximately one-third of participants felt they needed to lose weight; 40% of TYA cancer patients, TYA cancer survivors and controls did not think they needed to change their weight. Less than 10% of participants in each group thought they should drink less.

**Table 5:** Reported perception and change in current lifestyle since diagnosis

Reported perception of current lifestyle	TYA cancer patients % (*n*)	TYA cancer survivors % (*n*)	General population TYAs % (*n*)	Between group differences
Weight status				
I think I should be trying to lose weight	36.1 (30)	45.4 (79)	34 (109)	*p* =0.050
I think I should be trying to gain weight	14.5 (12)	10.3 (18)	10.6 (34)	
I don’t think I need to change my weight	41.0 (34)	40.2 (70)	40.8 (131)	
Don’t know	7.2 (6)	3.4 (6)	10.9 (35)	
Missing data	1.2 (1)	0.6 (1)	3.7 (12)	
Physical activity				
I think I should do more physical activity	70.7 (58)	71.1 (123)	67.0 (215)	*p* =0.141
I think I should do less physical activity	2.4 (2)	2.3 (4)	2.5 (8)	
I don’t think I need to change my physical activity	20.7 (17)	25.4 (44)	24.9 (80)	
Don’t know	6.1 (5)	0 (0)	4.4 (14)	
Missing data	0 (0)	1.2 (2)	1.2 (4)	
Diet				
I think I should have a healthier diet	55.8 (43)	53.2 (82)	53.3 (171)	*p* =0.151
I don’t think I need to change my diet	31.2 (24)	39.6 (61)	33.0 (106)	
Don’t know	9.1 (7)	4.5 (7)	10.6 (34)	
Missing data	2.6 (2)	1.9 (3)	3.1 (10)	
Alcohol consumption				
I think I should drink less	6 (5)	5.7 (10)	9.3 (30)	*p* =0.915
I don’t think I need to change my alcohol consumption	62.7 (52)	63.8 (111)	76 (244)	
Don’t know	8.4 (7)	6.9 (12)	9.3 (30)	
Missing data	21.9 (19)	23.6 (41)	5.3 (17)	
Change in lifestyle since cancer				
Weight status				
More than before cancer	50.6 (42)	59.2 (103)	–	*p* =0.245
About the same as before cancer	24.1 (20)	23.0 (40)	–	
Less than before cancer	24.1 (20)	17.8 (31)	–	
Missing data	1.2 (1)	0 (0)	–	
Physical activity				
More than before cancer	16.9 (14)	28.9 (50)	–	*p* =0.003
About the same as before cancer	14.5 (12)	25.4 (44)	–	
Less than before cancer	66.3 (55)	44.5 (77)	–	
Missing data	2.4 (2)	1.2 (1)	–	
Diet				
Healthier than before cancer	31.2 (24)	42.2 (65)	–	*p* =0.053
About the same as before cancer	44.2 (34)	44.2 (68)	–	
Less healthy than before cancer	22.1 (17)	11 (17)	–	
Missing data	1.6 (2)	2.6 (4)	–	
Alcohol consumption				
More than before cancer	8.4 (7)	27.0 (47)	–	*p* <0.001
About the same as before cancer	27.7 (23)	28.2 (49)	–	
Less than before cancer	41.0 (34)	20.1 (35)	–	
Missing data	22.9 (19)	24.7 (43)	–	
Smoking*	*n* =8	*n* =9		
Tried to quit since cancer diagnosis	50.0 (4)	44.4 (4)	–	*p* =0.539
Not tried to quit since cancer diagnosis	50.0 (4)	55.6 (5)	–	

*Only smokers included in analyses

### Change in health behaviour since diagnosis

Table 5 displays cancer patients and survivors reported change in health behaviour since diagnosis. Most young people with cancer reported that their physical activity levels were lower and their weight greater than before their diagnosis. The proportion of young people reporting making positive lifestyle changes (better diet and more physical activity) was greater among TYA cancer survivors who had finished cancer treatment (*p* ≤0.05) Forty-one percent (*n* =43) of TYA cancer patients reported that they drank less alcohol since their diagnosis. Of those who reported smoking (*n* =17), most were attempting to quit with approximately half indicating the quit attempt had been initiated following their diagnosis.

## Discussion

The primary aim of this study was to compare the health behaviours of young people with cancer to general population controls. Our results indicate that, despite the importance of health behaviour in cancer survivorship [27–29], young people with cancer in the UK have a similar health behaviour status to their peers in that they are largely inactive, consume diets low in fibre, lean meat and fruit and vegetables and regularly binge on alcohol. Nevertheless, reassuringly, a high proportion of TYA cancer patients and survivors held a desire to make positive lifestyle changes, specifically to be more active and have a healthier diet. Collectively, these results suggest that TYA cancer patient and survivors need, and would be receptive to, health behaviour change interventions.

The finding that the majority (>70%) of TYA cancer patients undergoing active treatment do not meet physical activity guidelines may in part be explained by limitations resulting from their cancer therapy such as hospitalisation, weakened immune system and high levels of fatigue [30, 31]. The lack of significant difference in physical activity between TYA cancer survivors and general population controls is reflective of a recent systematic review of studies comparing the physical activity levels of children and adolescents with type 1 diabetes, cardiovascular disease, or chronic respiratory disease and healthy population controls [32]. These consistent findings of no difference suggest young people with cancer make efforts to return to the same level of activity they had prior to their cancer diagnosis and that intrapersonal, interpersonal and environmental factors which explain low levels of physical activity among the general population apply equally to young people living with a chronic health condition [33]. Thought must therefore be given to whether existing interventions which have demonstrated positive effects on the physical activity levels of TYAs affected by other chronic conditions could be adapted for TYAs living with and beyond cancer.

Among both TYA cancer patients and survivors and controls, fruit and vegetable intake was strikingly poor with less than a quarter of young people meeting the current recommendations of five portions per day. The finding that most young people with cancer do not meet recommendations for fibre, fat or sugar intake is similar to previous studies which have investigated nutrition and dietary intake among childhood cancer survivors [34–36]. The high proportion of young people meeting current recommendations for red meat intake of no more than 500 g per week is most likely explained by the high intake of processed meat observed.

TYA cancer survivors have expressed a desire for lifestyle which is specific to the impact and effects of cancer treatment [37]. Several narrative reviews discussing dietary intake among childhood cancer survivors suggest young people with cancer crave energy dense foods high in sugar or salt during treatment [10, 38]. Although these cravings are thought to be an acute response to cancer therapies such as glucocorticosteroids, there is evidence that on completing treatment, young people have difficulty reversing unhealthy eating habits and often suffer lasting changes in taste preference which interfere with their ability to consume certain foods post treatment [39]. A similar phenomenon has been reported with regard to physical activity with parents and carers sometimes being overly cautious and discouraging young people being active during cancer therapy [31]. Data on physical activity and dietary intake gathered within this study emphasises the need for early intervention to promote healthy lifestyle choices from the point of diagnosis onwards.

The finding that almost a third of young people with cancer were obese or overweight and more than half indicated their current weight was greater than before diagnosis is concerning. However, it is unclear if weight gain observed among young people with cancer is due to the effects of cancer treatment, increased energy intake and/or decreased physical activity [40]. Research investigating whether treatment-related weight gain, weight loss and muscular atrophy could be mitigated or managed through effective diet and physical activity interventions is very much required [41, 42].

Encouragingly, very few young people within this study reported being either a current or former smoker and the number of smokers within this study was lower than the estimated proportion of childhood cancer survivors engaging in smoking [11]. Whilst there was no difference in the prevalence of smoking between young people with cancer and controls, high levels of alcohol consumption (particularly binge drinking) were observed in both groups. Moreover, a third of TYA cancer patients and survivors between 13 and 17 years of age reported under-age drinking. These data are reflective of The European School Survey Project on Alcohol and Other Drugs (ESPAD), which found 45% of males and 36% of females aged between 15 and 16 years in the UK, France, Finland, Denmark and Belgium, binge drink regularly [43]. Such patterns of binge, and under-age drinking, among young people are again a cause for concern as these behaviours are strongly linked to health and social problems [44, 45]. The finding that fewer TYA cancer patients reported binge drinking most likely reflects the ill health of young people with cancer during treatment and their social isolation away from friends and peers. Worryingly, very few young people reported a desire to change their levels of alcohol consumption, suggesting interventions explicitly targeting alcohol consumption are needed.

The overall non-significant difference in health behaviour status between TYA cancer survivors and controls may be because young people make positive changes to their health behaviour after their diagnosis and treatment. Twenty-eight percent of TYA cancer survivors reported that they had increased their levels of physical activity and 42% reported their diet was healthier than before cancer. These findings, combined with the finding that most TYA cancer patients and survivors felt they should be more active, eat a healthier diet and lose weight, support the hypothesis that a cancer diagnosis and its subsequent treatment may be a ‘teachable moment’ (i.e. an event which increases an individual’s motivation to improve their health behaviour). However, as demonstrated by the poor health behaviour status observed among both young people with cancer and general population TYAs, the adoption and maintenance of healthy lifestyle choices during and after cancer treatment is unlikely to occur without intervention.

This study has a number of limitations. Unfortunately, due to the small sample size, it was not possible to sub-classify cancer patients or survivors by cancer type or treatment exposure and therefore we could not establish if health behaviour status varies by diagnosis and therapeutic regimen. Future studies should investigate in closer detail the specific impact of these factors so that health behaviour interventions may be modified and adapted to meet the needs of young people of different cancer types and stages of treatment. Thought must also be given to when health behaviour interventions should be initiated among young people with cancer. The finding that the health behaviour status of TYA cancer patients undergoing treatment is poor suggests interventions introduced early during treatment or as a young person approaches the end of therapy may be beneficial in preventing the commonly observed declines in physical activity and dietary quality.

There were differences in the characteristics of young people with cancer compared to controls, with young people in the general population being significantly younger and more likely to be female. These differences, which limit the generalizability of the results, are most likely due to the recruitment methods. General population TYAs were mainly recruited via schools and higher education institutions whilst the recruitment of young people with cancer was highly targeted and conducted through UCLH and CLIC Sargent. As with most health research, response bias, recall bias and inaccuracies due to under and over reporting are likely to be present. It is therefore possible that the proportion of young people with cancer and general population TYAs meeting current health behaviour recommendations may be even lower than estimated.

## Conclusion

This study provides novel insight into the health behaviours of TYA cancer patients and survivors in the UK and how these compare to general population controls. Our data indicate that young people do not adhere to current health behaviour guidelines and engage in behaviours which have the potential to exacerbate their risk of ill health and future health problems. The finding that TYA cancer patients and survivors have a desire to change their health behaviour is encouraging and further strengthens the rationale behind a specifically designed health behaviour intervention for young people with cancer. Health professionals working with young people with cancer should recognise that their patients need, and would be open to receiving, support to change their health behaviour.
